# How cultural competence is conceptualised, developed and delivered in pharmacy education: a systematic review

**DOI:** 10.1007/s11096-023-01644-3

**Published:** 2023-09-27

**Authors:** Rawand Jarrar, Rosemary Lim, Charlotte Lucy Richardson, Atta Abbas Naqvi, Adam Pattison Rathbone, Wing Man Lau

**Affiliations:** 1 School of Pharmacy, Faculty of Medical Sciences, Newcastle University, King George VI Building, Newcastle upon Tyne, NE1 7RU UK; 2 Reading School of Pharmacy, University of Reading, Whiteknights, Reading, RG6 6AP UK

**Keywords:** Cultural awareness, Cultural competence, Cultural sensitivity, Pharmacist, Pharmacy, Pharmacy education

## Abstract

**Background:**

It is important to have a pharmacy workforce that is culturally competent to recognise a patient’s health beliefs to improve medication adherence and reduce poor treatment outcomes.

**Aim:**

This systematic review aimed to identify, critically appraise and summarise how cultural competency is conceptualised, developed and embedded in pre-qualification pharmacy education.

**Method:**

Medline, Scopus, PsychInfo, Web of Knowledge, CINAHL, and Embase databases were searched for relevant papers published in English between January 2012 and December 2021, following PRISMA guidelines. Data from included papers were thematically analysed. Educational quality of papers was appraised using the GREET criteria. This systematic review was registered on PROSPERO, CRD42021295875.

**Results:**

The review included 47 papers (46 studies) with 18 papers meeting ≥ 9 points on the GREET criteria thus considered of good educational quality. Forty papers focused on educational interventions implemented to pharmacy students only, the remaining included students from different health disciplines. Half of the educational interventions focused on cultural competence in general. Most educational interventions lasted over a week and 21 were compulsory. Cultural competence conceptualisation varied; a focus on knowledge about different cultures or on culturally competent behaviours or a continuum with knowledge at one end and behaviour at the other.

**Conclusion:**

There is variation in how cultural competence is embedded in pharmacy programmes, which could be a reflection of the differences in how educators conceptualised cultural competence. Further research is needed to develop a unified understanding of the meaning of cultural competence and how it can be embedded in pharmacy education.

**Supplementary Information:**

The online version contains supplementary material available at 10.1007/s11096-023-01644-3.

## Impact statements

- The evidence presented in this systematic review provides an understanding of how culture competence is taught to pre-qualification pharmacy students.
- Future educational intervention studies should be reported in compliance with an accepted set of reporting standards to facilitate quality assessments, replicability and validity.
- This review indicated a unified definition of cultural competence is needed so educators can understand how it should be taught to pharmacy students.

## Introduction

Culture refers to the values, beliefs and norms that are adopted by a specific group which guide thinking and behaviour. Culture can relate to ethnicity, age, gender, groups with special needs, religion, socioeconomic status, sexual orientation, and health beliefs [1, 2]. Culture is unconscious and affects all aspects of life, including experiences of health and illness. The influence and impact of culture on health is complex and not always understood, but there is a need to consider culture when providing care [1–4]. With increasing diversity of populations due to globalisation, the need for culturally competent health professionals has increased [4, 5]. Failure to address cross-cultural issues in the delivery of healthcare services can reduce patient satisfaction and compromise health outcomes [4, 5].

Cultural competence (CC) has been defined in several ways. One of the earlier definitions by Cross [6] states CC is: *‘a set of congruent behaviours, attitudes, and policies that come together in a system, agency, or amongst professionals and enables that system, agency, or those professionals to work effectively in cross-cultural situations’*. Campinha-Bacote [7] defined it as: *‘an ongoing process in which the healthcare provider continuously strives to achieve the ability to effectively work within the cultural context of a client’* [1, 8]. The literature indicates there may be different ways CC can be understood, which presents a challenge for the teaching and training of healthcare professionals.

As integral members of the healthcare team, pharmacists are medicines experts tasked with the delivery of pharmaceutical care [8]. Pharmaceutical care relates to medication supply but also how the medication is used and can be influenced by non-clinical patient-related factors, such as culture, socioeconomic status and language [8]. Failure to accommodate a patient’s health beliefs, could lead to poor treatment adherence and reduced health outcomes [1]. In line with the General Pharmaceutical Council’s Standards for pharmacy professionals in the UK and the Accreditation Standards and Guidelines for the Professional Program in Pharmacy in the US, when considering a patient’s culture, pharmacists should not stereotype by applying cultural characteristics from a specific culture to all patients, as this can lead to inappropriate healthcare decision making [1, 9]. Rather, pharmacists should “*recognise and value diversity, and respect cultural differences—making sure that every person is treated fairly whatever their values and beliefs*” [10]. The Pharmacy Council of New Zealand (PCNZ) and the Truth and Reconciliation Commission of Canada also call for pharmacists to demonstrate CC skills and knowledge [11, 12]. However, there is no specific guidance on how to implement training that would help develop a culturally competent pharmacy workforce.

The development of a culturally competent health workforce requires the integration of cultural competency training in educational programmes. However, before doing so, a clear definition of the concept needs to be established. A recent systematic review identified tools used to assess cultural competency within pharmacy programmes and acknowledged considerable variation in the tools used to assess CC as the intended learning outcomes, design and target audience of the educational interventions differed [13]. To our knowledge, there are no systematic reviews exploring what CC means in pharmacy education, how it is conceptualised and as a consequence how it is embedded within pharmacy education.

### Aim

To identify, critically appraise and summarise how CC is conceptualised, developed and embedded in pre-qualification pharmacy education.

## Method

The protocol was registered with PROSPERO on 7th December 2021, Reference CRD42021295875 [14] in accordance with the PRISMA guidelines [15].

### Inclusion criteria

Studies were considered eligible for inclusion if they were:

(i) Published between 1st of January 2012 and 31st of December 2021 so as to include literature most relevant to contemporary pharmacy education.
(ii) Published in a peer-reviewed journal.
(iii) Written in English.
(iv) Described or explained educational interventions (or alternative terms with the same meaning) to improve cultural competency in pre-qualification pharmacy education.

Conference papers, abstracts, book chapters, dissertations, literature reviews and systematic reviews were excluded. Studies that focused on CC in postgraduate or post-registration pharmacy education or in areas other than pharmacy education were excluded.

### Search strategy

A systematic search was conducted between December 2021 and January 2022 on six databases: Medline, Scopus, PsychInfo, Web of Knowledge, Cumulative Index of Nursing and Allied Health Literature (CINAHL) and Embase. The full search terms and key words used are shown in Supplementary material 1. Search results were exported to the Rayyan systematic review application [16] for screening and to remove duplicate papers. Reference lists of included papers and relevant systematic reviews on CC were screened for additional papers. Searches were carried out by author RJ and quality checked by RL and WML. Screening of titles and abstracts were conducted by authors RJ, WML, RL, and a random sample of 20% were cross-checked by RL and WML. Screening of full texts for relevance was done by RJ and WML with a further 20% cross-checked by RL. Any discrepancies were resolved by discussion between authors until consensus was reached.

### Data extraction process

An initial data extraction form was drafted by author RJ and piloted on 10 included papers. RL and WML revised the data extraction elements and the initial extraction. The initial form was modified based on initial reflections to create the final extraction form (Supplementary material 2). Extracted information included: authors and year, study title, study objectives, country of study, methods for data collection and analysis, terms and definition(s) of CC, methods for integrating CC in pharmacy education programme, year/level where educational interventions were implemented, and the outcomes of the educational interventions. Data extraction was carried out by RJ and 20% was cross-checked by WML and RL with no disagreement.

### Risk of bias and quality assessment

Risk of bias was assessed using the Mixed Methods Appraisal Tool (MMAT) (Supplementary material 3) [17]. All studies were evaluated by RJ and 20% (10 papers) was independently evaluated by RL [18]. Studies were not excluded based on the MMAT results because the focus of the review was on the nature of the interventions rather than their effectiveness.

The guideline for reporting evidence-based practice educational interventions and teaching (GREET) checklist which contains 17 criteria [19] was used to evaluate the quality of the educational intervention reporting. Criteria were attributed 1 or 0 points for compliance or non-compliance, respectively, and the total score for each paper was then calculated. The final quality scores were categorised arbitrarily by the authors as follows: a score ≥ 9 points (50%) indicated good quality and a score < 9 was considered low quality. Author AAN assessed the included papers using the GREET checklist and 20% was cross-checked by RL.

### Data synthesis

Extracted data were uploaded to NVivo where authors RJ, APR, AAN thematically analysed the data using the method outlined by Thomas and Harden [20]: 1) identify study characteristics, 2) identify study findings in descriptive themes that capture and summarise the findings of the literature, 3) identified analytic themes, to go beyond the original findings of the literature and add new knowledge. A deductive synthesis was used throughout, which focused on conceptualisations of CC and how CC teaching was embedded and delivered within pre-qualification pharmacy education curricula. The synthesis process was iterative and included discussions with WML, RL and CR.

## Results

### Paper selection

**Fig. 1:**
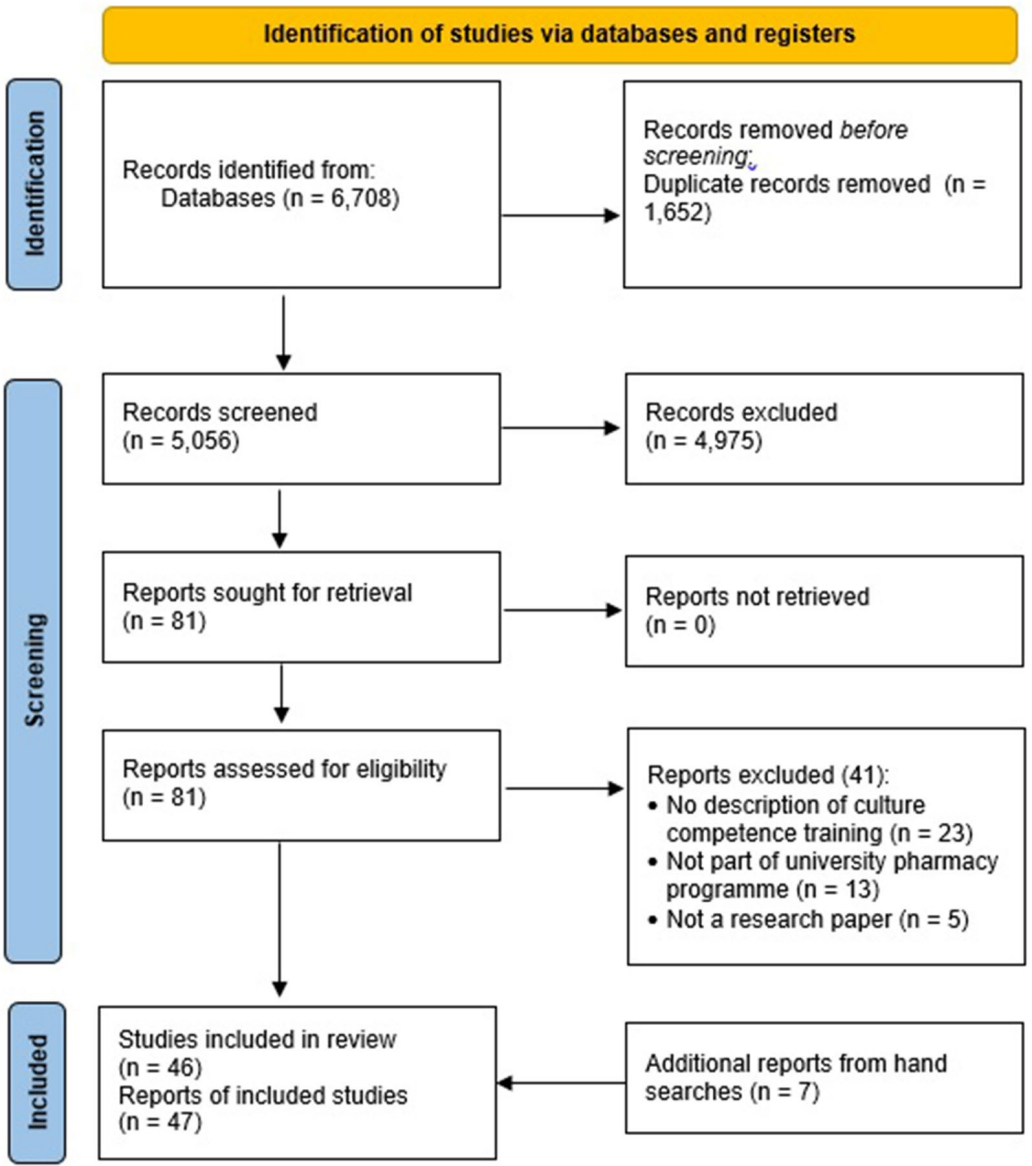
PRISMA diagram

The paper selection process is summarised in the PRISMA Diagram (Fig. 1). The search identified 6,708 records. After the removal of duplicates, 5,056 papers were retrieved and included in the screening of titles and abstracts which excluded 4,975 papers. Eighty-one papers were included in the full text review with 40 papers remaining for inclusion. Seven additional papers were found for inclusion after screening the reference lists of relevant systematic review papers and included studies. A total of 47 papers (46 studies) were included.

### Study characteristics

Forty papers assessed educational interventions to teach CC and six papers reported frameworks for teaching CC in pharmacy programmes. Forty papers focused on pharmacy student only as research participants, whilst the remaining seven included students from multiple healthcare disciplines (including pharmacy). Most papers were conducted in the United States (n = 39), followed by Aotearoa New Zealand (n = 2), Canada (n = 2), Germany (n = 1), Qatar (n = 1), United Arab Emirates (n = 1) (Table 1).

**Table 1:** Characteristics of included studies

Author(s)	Country	How intervention was delivered	Elective or compulsory course	Year(s) of study	Target student group	Focus	Delivery method	Duration of intervention	Number of students
Arif et al. [57]	US	Integration in Health communications course	Part of compulsory course	3	Pharmacy only	Cross-cultural communication	Lectures and workshops Patient video vignettes	5 h: 3 h of lecture and 2 workshop hours	159
Arif et al. [58]	US	standalone course	Elective course	2,3	Pharmacy only	Influence of culture on disease states	Lectures, workshops, simulations, and community work	6 weeks; 1 h lectures followed by half-hour workshops	31
Aspden et al. [59]	New Zealand	Intervention/ event	Compulsory	2	Pharmacy only	Perceptions of people living in poverty	Simulation exercise	3-h exercise	79
Aspden et al. [60]	New Zealand	Conceptualisation	NA	All	Pharmacy only	Cultural competence in general	NA	NA	NA
Bailey et al. [43]	US	Standalone course	Elective course	1, 2, 3	Pharmacy only	Communicating with deaf and hard of hearing patients	Lectures and practice sessions	4 classes (90 min) and additional practice sessions	39
Boylan et al. [26]	US	Integrated in non-prescription medication course	Compulsory for students enrolled in the course	1	Pharmacy only	Use of complementary and alternative medicine	Preparing 10 min presentations by students	Students prepared presentation over 6 weeks	80
Butler et al. [21]	US	Activities integrated in different courses	Varied	1, 2, 3	Pharmacy only	Cultural competence in general	Classroom activities	one class per year	1009
Cailorand Chen [35]	US	Integrated in 3 courses: Self-Care, Introduction to Pharmacy Practice, and Pharmacy Practice Lab (skills lab)	Compulsory	1	Pharmacy only	Cultural competence in general	Practical activities and discussion	One semester	53
Chen et al.[36]Addition to Cailordand Chen [35]	US	Integrated in capstone course, practice experience and disease modules	Compulsory	All	Pharmacy only	Cultural competence in general	Practical activities and discussion	Year 1: 9 h Year 2 and 3: more than 6 h/year + 2 h in capstone course. Year 4: in practice experience	189
Chen. et al. [44]	US and Canada	Survey on cultural competency implementation	Varied	All	Pharmacy only	Cultural competence in general	NA	NA	NA
Clarke et al. [27]	US	Activity as part of IPPE	Compulsory	2	Pharmacy only	Perceptions of people living in poverty	Simulation exercise	3 h	108
Cooper et al. [61]	US	Integrated in IPPE	Compulsory	4	Pharmacy only	Cultural competence in general	Lectures	6 h	194
Crawford et al. [38]	US	Integrated in different courses	Compulsory	All	Pharmacy only	Culture awareness and culture sensitivity	Lectures, discussions, and workshops	Lectures were dispersed throughout curriculum	656
Diaz-Cruzand Hagan [48]	US	Integrated in the orientation programme	Elective course	1	Pharmacy only	Cultural Proficiency Continuum Framework	Lecture and group discussion	90 min	100
Durand et al. [62]	US	standalone course	Elective course	Not mentioned	Pharmacy only	Cultural competence in general	Lectures and Hands-on experiences	3h/week for 10 weeks	12
Dushenkov et al. [28]	US	Integrated in the capstone course	Compulsory	4	Pharmacy only	Cultural competence in general	Workshop	Workshop including lecture and videos	164
Echeverri and Dise [63]	US	Assess student cultural competence profiles	NA	1,2	Pharmacy and medicine	Cultural competence in general	Lectures, group sessions, independent research, practical experiences, and online training	Year 1: 2 × 1-h lectures, small group session, semester long experience year 2: 3-h group session, 4 h online training, participation in activities	539 (285 pharmacy)
Gibson and White [47]	US	Integrated in Pharmacotherapy course	elective activity	4	Pharmacy only	Culture sensitivity to special populations	Panel discussion	1 h in 2016 2 h in 2017	69 in 2016 78 in 2017
Haack and Phillips [64]	US	Integrated in Pharmacy Skills and Application course series	Compulsory	All	Pharmacy only	Cultural competence in general	Lectures and laboratory activities	dispersed throughout the programme	206
Hasan et al. [22]	UAE	Integrated in communication skills and counselling course	not mentioned	3	Pharmacy only	Language training	Lecture, role play, pre-tutorial homework	13 weeks	72
Hawala-Druy and Hill [29]	US	standalone course	Elective course	Not mentioned	Pharmacy, Nursing and Allied Health Sciences	Cultural competence in general	Lectures, outside activity, and practical experience	3 h/week for 14 weeks	106 (42 pharmacy)
Hefferman et al. [49]	US (Hawaii)	Standalone field trip	elective activity	All	Pharmacy only	cultural implications of Hansen disease in Hawaii	Independent research, field trip, and group discussions	One day trip	18
Johnson and Trynor [30]	US	Explore required learning competencies to work in underserved populations	NA	NA	Pharmacy only	Working with underserved patients	NA	NA	NA
Knockel et al. [65]	US	Integrated in Applications of Pharmacy Practice I course	Compulsory	2	Pharmacy only	LGBTQ health	Lecture	One hour	107
Leach et al. [41]	US	Integrated in Endocrine, Women's Health, and Genitourinary	Compulsory	2	Pharmacy only	Transgender healthcare	Lecture	One hour	60
Liu et al. [39]	US	CC and IPE sessions as part of required course that focused on cultural competency and health literacy	Compulsory	2	Pharmacy and nursing	Cultural competence and interprofessional work	Team-based discussions of videos and case studies	2 sessions	160 (80 pharmacy)
Lucas et al. [24]	Australia	Insights about developing curriculum on cultural safety	NA	NA	Multiple health disciplines	Indigenous curriculum	NA	NA	NA
McKennon et al. [45]	US	Standalone course on Herbal Medicines and Natural Product Drugs	Compulsory	3	Pharmacy only	Herbal medicine and natural drugs	Lectures Assignments Case studies	One semester	59
Min et al. [25]	Canada	Standalone course in indigenous health	Elective	3,4	Pharmacy only	Indigenous health	Lectures Video conference Practical experiences Case studies	One term	101
Minshew et al. [46]	US	Create a cultural competence framework	NA	NA	Pharmacy only	Cultural intelligence	NA	NA	NA
Mueller [23]	US	Standalone course on medical Spanish	Elective	3	Pharmacy only	Medical Spanish	Lectures Simulated patient activities Guest presentation	One semester	4 in 2014 3 in 2015
Nebergall et al. [42]	US	Embedded in Integrated Patient Care Laboratory	Compulsory	1, 2, 3	Pharmacy only	Underserved populations	Lectures Skills laboratory Practical experience	40 h lectures and skills labs throughout semester One-week practical experience	119
Newsome et al. [66]	US	Integrated in Therapeutics of Special Populations course	Compulsory	3	Pharmacy only	Transgender healthcare	Active learning Patient cases Panel discussion	3 h	152
Okoro et al. [33]	US	Integrated in Professional Communications in Pharmacy Practice course	Compulsory	2	Pharmacy only	Cultural competence in general	Lectures	2 lectures (1.5 h in total)	2010 (n = 294) 2011 (n = 279) 2012 (n = 287)
Ostroff et al. [32]	US	Integrated in special populations course	Compulsory	3	Pharmacy only	Transgender healthcare	Lecture Assignments Video screening	2 h	72
Parkhill et al. [40]	US	Integrated in the Introduction to Diversity Course	Compulsory	1	Pharmacy only	Transgender healthcare	Panel discussion	2 h	Not mentioned
Prescott and Nobel [50]	US	Integrated in Pharmaceutical care I course	Compulsory	1	Pharmacy only	Cultural competence in general	Lecture and practical session: discussion, video, counselling	110-min lecture and 4-h practicum	136
Rovers et al. [67]	US-trip to Dominican Republic	Included in global health experience	Elective	Not mentioned	Multiple health disciplines	Cultural competence in general	International practical experience	One week	20 (2 pharmacy)
Schellhase et al. [68]	US	Standalone course	Elective	3	Pharmacy only	Competencies for international experiences in Kenya	Lectures Case studies Discussions	15 weeks	2011 = 26 2012 = 24
Scott et al. [69]	US	Included in global health experience	Elective	All	Pharmacy only	Cultural competence in general	Study abroad: seminars, lectures, practical experience, and culture events	3 weeks	13
Sheu et al. [70]	US	Standalone practice experience	Elective	1	Medical, nursing, and pharmacy students	Health disparities and CC	Student-run clinics: didactic sessions and volunteering	Various	358 (pharmacy not specified)
Steeb et al. [71]	US	Standalone practice experience	Elective	4	Pharmacy only	Global health	International advanced pharmacy practice experience (APPE)	4–8 weeks	Not mentioned
Strelow et al. [34]	Germany	Integrated in a seminar series	Elective	2, 3	Medicine, pharmacy, and translation	Communities who speak different languages	Lectures, discussions, and practical training days	one joint day and two practical days	112 (60 pharmacy)
Thomason et al. [72]	US	Part of practice experiences	Compulsory	4	Pharmacy only	Underserved populations	Practice experience	one month or more	122
Werremeyer and Skoy [73]	US-trip to Guatemala	Standalone practice experience	Elective	3	Pharmacy only	Cultural competence in general	Practice experience	5 weeks	4 students: 2 in 2010 and 2 in 2011
Wistholter et al. [37]	US- experience in South Africa	Standalone practice experience	Elective	All	Pharmacy only	Cultural competence in general	Practice experience	4 weeks	3–4 students per year
Wilby et al. [74]	Canada and Qatar	Integrated in different courses	Elective	Canada: 2 Qatar: final	Pharmacy only	Cultural competence in general	Discussion via videoconference	2 h	22 in Canada and 22 in Qatar

### Quality of included papers

The results of the risk of bias assessment using MMAT tool is reported in Supplementary material 3. All the papers had clear research questions and the data collected addressed the research questions. However, for quantitative studies and mixed-methods studies, there were more papers with unclear reporting of sampling and risk of bias.

The educational quality of the studies was assessed using the GREET checklist [19]. Seven paper were not assessed as they reported educational frameworks (Supplementary material 4). Of the 40 included papers, 18 were of good quality (complied with > 9 criteria). All papers complied with item 1 by providing a description of the education intervention for the participants involved. 25 papers (62.5%) complied with item 3 by describing the learning objectives, but none recorded information about the process used to determine that the teaching materials/strategies/sessions were delivered as originally planned (item 16 and 17).

### Characteristics of CC educational interventions

This section summarises the education, teaching and training interventions reported in the literature and how they were delivered in pre-qualification pharmacy education.

1. What is done?
  Educational intervention topics included different languages, cultures, ethnicities, religions practices, sexualities and groups with special needs. Most of the institutions that hosted research activities were based in the US (n = 39). Others involved international placements in the Dominican Republic, Guatemala, Kenya, and South Africa (Table 2).

**Table 2:** Focused topics and cultures of interventions

	N
Focused topics, if mentioned^a^	
Cross-culture healthcare, sensitive language, interpreting	20
Diversity, cultural sensitivity, and awareness	18
Socioeconomic factors e.g., financial and housing instability, social determinants of health	14
Medication use e.g., drug abuse, overdose	8
Bias, Generalization, and stereotypes	4
Legal, ethical, insurance, professional obligations	4
Race/racial and ethnic disparities	3
Social identity	3
Use of herbal products in Latin America	3
Mental health	2
Disability	1
Family care givers	1
Inter-professional collaboration	1
Organ donation	1
Focused culture if mentioned^a^	
Region/culture specific	
Latin American/Latino culture	2
Cultures of the Asian/Pacific Islands	1
Bosnian culture	1
Dominican culture	1
French Canadian	1
German culture	1
Ghanian culture	1
Guatemalan Communities	1
Hawaiian culture	1
Igbo culture (Nigerian region)	1
Indigenous communities	1
Italian culture	1
Kenyan culture	1
Maori culture	1
Nigerian culture	1
North American and Irish	1
Southeastern Americans	1
Spanish speakers	1
Religion	
Christianity	1
Judaism	1
Patient groups	
LGBTQ +	5
Special/vulnerable populations e.g., South African patients with HIV	5
Underserved populations	5
Hard of hearing and visually impaired patients	3
People who smoke	1

^a^Categorisation is not mutually exclusive
  Educational interventions were evaluated using a range of learning outcome measures. ‘Attitude’ was most used outcome measure (n = 29), followed by ‘knowledge’ (n = 24), and ‘skills’ (n = 18) (Table 3). There was variation in the methods used to collect and analyse the effect of educational intervention, which means it is not possible to compare interventions directly. Of the 41 papers, only 2 papers reporting no difference in outcomes measures following the educational intervention. Further work may be needed to encourage researchers to disseminate findings transparently, including negative and neutral findings.

**Table 3:** Summary of learning outcomes assessed and effect of cultural competence educational interventions

	Author(s)	Learning outcomes assessed								Effect
		Awareness	Knowledge	Attitude	Skills	Encounters	Desire	Practice	Overall competence	
1	Arif et al. [57]		+	+						+ / +
2	Arif et al. [58]		+	?	+					+ /?/ +
3	Aspden et al. [60]			?						?
4	Bailey et al. [43]			+						+
5	Boylan et al. [26]			0						0
6	Butler et al. [21]		+	0	+	+				+ /0/ + / +
7	Cailor et al. [35]	+	+		+	+	+		+	+ / + / + / + / + / +
8	Chen et al. [36]	+	+		+	+	+			+ / + / + / + / +
9	Clarke et al. [27]			?						?
10	Cooper et al. [61]			?						?
11	Crawford et al. [38]		+	0	+	+				+ /0/ + / +
12	Diaz-Cruz et al. [48]		+							+
13	Durand et al. [62]	+	+		0	+	+		+	+ / + /0/ + / + / +
14	Dushenkov et al. [28]		?	+						?/ +
15	Echeverri and Dise [63]	?	?	?	?	?			?	?/?/?/?/?/?
16	Haack and Phillips [64]	0	0		+	+	0			0/0/ + / + /0
17	Hasan et al. [22] †			+	?					+ /?
18	Hawala-Druy and Hill [29] †			?					+	?/ +
19	Heffernan et al. [49] ‡		+	+	+					+ / + / +
20	Knockel et al. [65]		+	+						+ / +
21	Leach et al. [41]			+						+
22	Liu et al. [39]		+	+	+	+				+ / + / + / +
23	McKennon et al. [45]			?						?
24	Min et al. [25] †	+	+	+				+		+ / + / + / +
25	Minshew et al. [46] ‡	+	+				?	0		+ / + /?/0
26	Mueller [23]				+					+
27	Nebergall et al. [42] †			+	+					+ / +
28	Newsome et al. [66]			+						+
29	Ostroff et al. [32]		+	+						+ / +
30	Parkhill et al. [40]		?	?						?/?
31	Prescott and Nobel [50]		+	+	+					+ / + / +
32	Rovers et al. [67]	?	+		+	?	?		+	?/ + / + /?/?/ +
33	Schellhase et al. [68]		+							+
34	Scott et al. [69]		+	0						+ /0
35	Sheu et al. [70]			0						0
36	Steeb et al. [71]	+	+	+	+				+	+ / + / + / + / +
37	Strelow et al. [34]				?					?
38	Thomason et al. [72]			?						?
39	Werremeyer and Skoy [73]								+	+
40	Wietholter et al. [37]		+	+	?					+ / + /?
41	Wilby et al. [74]			+						+

NB: The following symbols are used to denote the effects shown on the learning outcome: + beneficial effect., ? unclear or mixed effect, 0 no effect,—negative effect, † mixed-methods study, ‡ qualitative study
2. How is it done?
  Several approaches to deliver teaching were reported, such as lectures, workshops, group activities and placements (Table 4). Most papers (n = 35) used didactic modes of teaching, either independently or combined with other activities, such as discussion groups. Most (n = 24) reported educational interventions lasting longer than one week, with the remaining papers reporting contact time of less than one day (n = 14).

**Table 4:** Delivery of interventions

	N
*Mode of intervention*^a^	
Didactic	35
Field trips and experiential training e.g., community immersion and patient engagement	32
Discussion-based e.g., case-based learning, team-based learning	24
In-person simulation	12
Assessment	6
Online learning activity (including online simulation)	5
Inter-professional work	2
*Contact time of intervention*	
≤ 8 h or 1 day	14
2–7 days	2
> 1 week	24

^a^Categorisation is not mutually exclusive
  An integrated approach to delivering CC curricula was reported (n = 25), where content was delivered as an element within a wider module e.g. modules concerning disease pathophysiology, therapeutics or wider aspects of public health. Some (n = 14) reported delivery of CC content within a stand-a-lone CC module. Additionally, approximately half (n = 21) reported that CC curricula was compulsory or part of a compulsory module, whilst 18 reported the content was covered within elective modules and one reported that it was delivered as part of compulsory and/or elective modules [21]. Hasan’s study incorporated CC as part of communications skills and counselling teaching, but did not specify whether participation was compulsory [22]. No reports included inter-professional collaboration with disciplines outside pharmacy (e.g. medicine). Collectively, this finding demonstrates heterogeneity of modes and duration of CC education in pharmacy.

### Conceptualisation of cultural competency in pharmacy education

The terms and models in the literature to describe CC and their application varied. Findings are presented below, which identify ‘how’ CCs was conceptualised, as a knowledge-based (Theme 1), skill-based (Theme 2) or behaviour-based (Theme 3) construct. Although CC was the most commonly used term (n = 22), several alternative terms including culture sensitivity, culture humility, culture intelligence, and culture proficiency were also used. These terms were either used interchangeably with CC or were chosen by some authors to emphasise a certain aspect of a wider spectrum.

- Theme (1) Knowledge-based
  - This theme focused on knowledge about specific cultures, such as language for Spanish-speakers, slang for Aboriginal Australians, historical events for First Nation Canadians, or complementary and alternative medicines used in certain cultures [23–26]. This proposition focused on knowledge about differences in constructs between cultures at an individual, familial and community level, which enabled students to recall and recognise specific cultures. The focus was not adapting one’s own behaviours but knowing the cultural needs of another and how to meet those needs, for example, by using an interpreter or recognising the importance of non-verbal communication.
    In these studies the cognitive conceptualisation of CC drew on other knowledge-based aspects of the curriculum, such as the Social Determinants of Health model [27–32], Patient Safety parameters [33, 34], decision making processes [35–37] and principles of patient-centred care [22, 34, 37–40]. Improving patient outcomes and reducing health disparities were also aspects considered by several studies [22, 27, 29, 30, 33, 34, 41, 42].
- Theme (2) Skill-based
  - Cultural sensitivity was another commonly used term; several studies referred to culture sensitivity as a standard skill set in Centre for the Advancement of Pharmaceutical Education (CAPE) and Accreditation Council for Pharmacy Education (ACPE) [27, 28, 33, 42–46]. In some instances, cultural sensitivity was not defined or compared with the concept of CC. When a definition was provided, it indicated cultural sensitivity entails the recognition of social determinants of health, adaptability to patients’ cultural beliefs without stereotyping, and providing a healthcare plan that considers patients’ own culture while being able to communicate effectively [28, 47]. While it seems cultural sensitivity might be synonymous with CC, some studies indicated there is a difference and cultural sensitivity could be a part of becoming culturally competent [21, 34, 48]. We suggest that these studies demonstrate cultural sensitivity as an action or skill, which creates opportunity for students to ‘show how’ they can be culturally sensitive and are working towards being culturally competent.
- Theme (3) Behaviour-based
  - Studies also referred to models conceptualising CC as a continuum from cognitive factors, for example knowing about other cultures, languages and what is important to people from a given culture, to behavioural factors, such as asking patients about their individual, familial or community cultural needs within everyday practice [28, 29, 38, 39]. This conceptualisation reflects a broader transformational behaviour change, which continually adapts through processes and perceptions. Diaz-Cruz [48] used the term ‘cultural proficiency’ and described this as an adaptation to differences in values and practices requiring personal transformation; the authors described that pharmacists can only strive for the goal of becoming culturally proficient, as this is a never-ending process of learning about and with cultures, where full competence cannot be achieved [48]. This conceptualisation positions CC as behaviours which are performed and practiced over time.

## Discussion

### Statement of key findings

Overall, the findings show CC was conceptualised as knowledge-, skill- and behaviour-based constructs. Conceptualisations of CC in the literature was heterogeneous with different terms, outcomes, modes and durations of teaching and training implemented in pre-qualification pharmacy education. Although most studies used the term ‘cultural competence’ to guide their educational interventions, variations in conceptualisation and terminology[25, 46, 49, 50], made it unclear if the learning outcomes should be knowledge-, skill- or behaviour-based, which can potentially mean differences in the design of educational interventions. Therefore, it would be difficult to understand and compare the effects of those interventions. This has further implications for other educators who wish to design and implement CC interventions for their own programme. Specific terms to describe different conceptualisations of CC are needed to reflect the spectrum from a knowledge-based learning outcome where they know about different cultures, towards behavioural capability outcome, which enables them to adapt to different cultures in their practice [46, 48, 51].

### Strengths and limitations

A robust and systematic approach was used to search, identify, screen, and evaluate literature for this review in line with the PRISMA guidelines [15]. Although screening, data extraction and quality assessment was independently conducted by one author, to ensure robustness of the review, one to two other authors were involved in assessing a sample of selected papers at each stage in order to determine whether further samples would need to be checked. Another limitation was the inclusion of only papers written in English, which may have inadvertently excluded relevant papers published in other languages. Grey literature was also excluded, which is often used to disseminate pedagogical research.

### Interpretation of findings

**Fig. 2:**
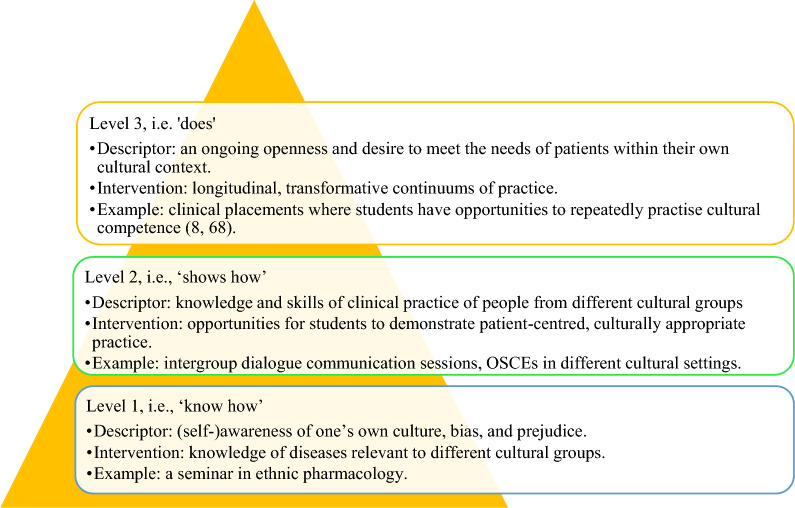
Conceptual model of cultural competence in pharmacy education

There is variation in the way CC is conceptualised in pharmacy education resulting in diverse CC educational interventions. This variation aligns with Miller’s triangle of competence that maps development from ‘knows’ to ‘does’ and is commonly used in pharmacy education and practice standards. Mapping our findings with Miller’s triangle [52], Campinha-Bacote’s [7] and Well’s model of CC [53], provides a model of conceptualisation of CC in pharmacy education (Fig. 2).

This variations can also be found in nursing and medical education [54, 55]. Cai [56] also found ambiguity of the definition of CC used in nursing contributed to variation in the instruments used to measure CC and thus made it difficult to assess their effectiveness.

### Future research

Almost half of studies were non-compliant to the GREET checklist for reporting educational interventional studies [19]. Although most studies were conducted before the GREET checklist publised, poor reporting of methods to document educational interventions may limit transferability. Future work should report educational interventions in compliance with an accepted set of standards, to facilitate quality assessment, replicability and validity.

In addition, most studies evaluated the effect of educational interventions on a short-term basis. Studies that reviewed the longitudinal impact of educational interventions reported a reduction in CC scores assessed after a lengthier gap [35]. This highlights that CC educational interventions designed in most studies cater to short-term competence needs of students and may not necessarily address the long-term learning needs or have a sustained impact on practice. Further research is needed into the longer-term implementation of educational interventions and the assessment of the effectiveness, especially given the heterogeneous outcomes currently measured.

## Conclusion

There is variation in how CC is taught and assessed in pre-qualification pharmacy education. Heterogeneity could be a consequence of differences in conceptualisation of CC. Therefore, further research is needed to develop a unified understanding of CC and how it should be taught to pharmacy students.

### Supplementary Information

Below is the link to the electronic supplementary material.

Supplementary file1 (DOCX 90 KB)
