# Supplementary material for: How cultural competence is conceptualised, developed and delivered in pharmacy education: a systematic review
Source: Int J Clin Pharm. 2023 Sep 27;46(1):40–55. doi: 10.1007/s11096-023-01644-3 (PMC10830822; doi:10.1007/s11096-023-01644-3)
Supplement: Supplementary file 1 — Supplementary file1 (DOCX 90 KB) [file 11096_2023_1644_MOESM1_ESM.docx]

**How cultural competence is conceptualised, developed and delivered in pharmacy education: a systematic review**

**Supplementary information**

**Supplementary material 1: Database search strategy**

| Cultur* competenc* OR cultur* humility OR cultur* sensitive* OR cultur* proficien* OR cultur* safety OR cross-cultur* OR cross cultur* OR de-colonis* OR decolonis* OR de-coloniz* OR decoloniz* OR Care, Culturally Competent OR Culturally Congruent Care OR Care, Culturally Congruent OR Culturally Competent Health Care OR Cross-Cultural Care OR Care, Cross-Cultural OR Cross Cultural Care OR Cultural Care OR Care, Cultural OR trans-cultur* OR transcultur* | AND | Concept OR concepts OR conceptualisation* OR conceptualization* OR define* OR definition* OR refer OR refers OR term* OR implement* OR integrat* OR embed* OR include* OR inclusion OR use* OR used OR uses OR utilise* OR utilize* OR utilization* OR utilisation* | AND | Education* OR train* OR training OR program* OR curriculum* OR module* OR teach* OR taught OR develop* OR student* OR trainee* OR graduate* OR degree* OR institution* OR undergrad* | AND | pharmacy OR pharmacist* OR pharmaceutical* |
| --- | --- | --- | --- | --- | --- | --- |

Search strategy completed on Medline, Scopus, PsychInfo, Web of Knowledge, Cumulative Index of Nursing and Allied Health Literature (CINAHL) and Embase.

**Supplementary material 2: data extraction form**

**Data extraction form for studies on culture competency education**

| **Study information** | |
| --- | --- |
| 1. Author: | 1. Year: |
| 1. Study title: |  |
| 1. Study objectives |  |
| 1. Geographical location: |  |
| 1. Context of the study: | *Guidelines or regulations surrounding cultural competences in the country where the study was conducted* |
| **Culture competence training description** | |
| 1. Culture competence components | *Culture competence concepts that were included in the course/training* |
| 1. Year level | *Programme year level of pharmacy students included in the programme* |
| 1. Number of participants in the course |  |
| 1. Participant characteristics |  |
| 1. Compulsory or elective course? |  |
| 1. Standalone course or integrated with other topics | *Whether students received the culture competence training as a standalone programme or whether it was integrated with other topics; e.g. global health or interprofessional education* |
| 1. Mode of delivery | *How the training was delivered; lectures, workshops, experience.... etc* |
| 1. Length of culture competence training |  |
| **Culture competence assessment** | |
| 1. Data collection method |  |
| 1. Data analysis method |  |
| 1. Number of participants included in the assessment |  |
| 1. Characteristics of participants included in the assessment |  |
| 1. Culture competence capabilities assessed |  |
| 1. Pre-training assessment results |  |
| 1. Post-training assessment results |  |

**Supplementary material 3: risk of bias assessment (MMAT tool)**

| QUANTITATIVE DESCRIPTIVE STUDIES | | | | | | | | |
| --- | --- | --- | --- | --- | --- | --- | --- | --- |
| Author/ | **Year** | **S1. Are there clear research questions?** | **S2. Do the collected data allow to address the research questions?** | **1. Is the sampling strategy relevant to address the research question?** | **2. Is the sample representative of the target population?** | **3. Are the measurements appropriate?** | **4. Is the risk of nonresponse bias low?** | **5. Is the statistical analysis appropriate to answer the research question?** |
| Arif et al | 2017 | Yes | Yes | Yes | Yes | Yes | Can't tell | Yes |
| Arif et al | 2019 | Yes | Yes | Yes | Yes | Yes | Yes | Yes |
| Bailey et al | 2021 | Yes | Yes | Yes | Yes | Yes | Can't tell | Yes |
| Boylan et al | 2020 | Yes | Yes | Yes | Yes | Yes | Can't tell | Yes |
| Cailor et al | 2015 | Yes | Yes | Yes | Can't tell | Yes | Can't tell | Yes |
| Chen et al | 2020 | Yes | Yes | Yes | Can't tell | Yes | Can't tell | Yes |
| Diaz-Cruz & Hagan | 2020 | Yes | Yes | Yes | Can't tell | Yes | Can't tell | Can't tell |
| Dushenkov et al | 2020 | Yes | Yes | Yes | Can't tell | Yes | Can't tell | Yes |
| Echeverri & Dise | 2017 | Yes | Yes | Yes | Yes | Yes | Can't tell | Yes |
| Haack & Phillips | 2012 | Yes | Yes | Yes | Can't tell | Yes | Yes | Yes |
| Knockel et al | 2019 | Yes | Yes | Yes | Can't tell | No | Can't tell | Yes |
| Mueller et al | 2017 | Yes | Yes | Yes | No | Yes | Yes | Can't tell |
| Newsome et al | 2018 | Yes | Yes | Yes | Can't tell | Yes | Can't tell | Yes |
| Okoro et al | 2015 | Yes | Yes | Yes | Yes | Yes | Can't tell | Yes |
| Ostroff et al | 2018 | Yes | Yes | Yes | Can't tell | Yes | Can't tell | Yes |
| Prescott and Nobel | 2019 | Yes | Yes | Yes | Can't tell | Yes | No | Can't tell |
| Schellhase et al | 2013 | Yes | Yes | Yes | Can't tell | Yes | No | Can't tell |
| Scott et al | 2019 | Yes | Yes | Yes | No | Yes | Can't tell | Yes |
| Strelow et al | 2021 | Yes | Yes | Yes | Can't tell | Yes | Can't tell | Can't tell |
| Wietholter et al | 2014 | Yes | Yes | Yes | Can't tell | Yes | Can't tell | Can't tell |
| Butler et al | 2020 | Yes | Yes | Yes | Can't tell | Yes | Can't tell | Yes |
| Chen et al | 2021 | Yes | Yes | Yes | Yes | Yes | Yes | Yes |
| Crawford et al | 2016 | Yes | Yes | Yes | Yes | Yes | Can't tell | Yes |
| Leach et al | 2019 | Yes | Yes | Yes | Can't tell | Yes | Can't tell | Yes |
| Liu et al | 2015 | Yes | Yes | Yes | Yes | Yes | Yes | Yes |
| Rovers et al | 2020 | Yes | Yes | Yes | Yes | Yes | Yes | Can't tell |
| Werremeyer & Skoy | 2012 | Yes | Yes | Yes | Can't tell | Yes | Yes | Can't tell |
| Durand et al | 2012 | Yes | Yes | Yes | Can't tell | Can't tell | Yes | Yes |
| McKennon et al | 2018 | Yes | Yes | Yes | Can't tell | Yes | Can't tell | Can't tell |
| Thomason et al | 2013 | Yes | Yes | Yes | Can't tell | Yes | No | Yes |
| Clarke et al | 2016 | Yes | Yes | Yes | Yes | Yes | Yes | Yes |
| Cooper et al | 2014 | Yes | Yes | Yes | Can't tell | Yes | Can't tell | Yes |
| QUALITATIVE STUDIES | | | | | | | | |
| Author | **Year** | **S1. Are there clear research questions?** | **S2. Do the collected data allow to address the research questions?** | **1. Is the qualitative approach appropriate to answer the research question?** | **2. Are the qualitative data collection methods adequate to address the research question?** | **3. Are the findings adequately derived from the data?** | **4. Is the interpretation of results sufficiently substantiated by data?** | **5. Is there coherence between qualitative data sources, collection, analysis and interpretation?** |
| Aspden et al | 2017 | Yes | Yes | Yes | Yes | Yes | Yes | Yes |
| Hefferman et al | 2013 | Yes | Yes | Yes | Yes | Yes | Yes | Yes |
| Lucas et al | 2021 | Yes | Yes | Yes | Yes | Yes | Yes | Yes |
| Johnson & Traynor | 2018 | Yes | Yes | Yes | Yes | Yes | Can't tell | Can't tell |
| Minshew et al | 2021 | Yes | Yes | Yes | Yes | Yes | Yes | Yes |
| MIXED-METHODS STUDIES | | | | | | | | |
| Author | **Year** | **S1. Are there clear research questions?** | **S2. Do the collected data allow to address the research questions?** | **1. Is there an adequate rationale for using a mixed methods design to address the research question?** | **2. Are the different components of the study effectively integrated to answer the research question?** | **3. Are the outputs of the integration of qualitative and quantitative components adequately interpreted?** | **4. Are divergences and inconsistencies between quantitative and qualitative results adequately addressed?** | **5. Do the different components of the study adhere to the quality criteria of each tradition of the methods involved?** |
| Min et al | 2020 | Yes | Yes | Yes | Yes | Can't tell | Can't tell | Can't tell |
| Nebergall et al | 2021 | Yes | Yes | Yes | Can't tell | Yes | Can't tell | Can't tell |
| Parkhill et al | 2014 | Yes | Yes | Yes | Yes | No | Can't tell | Can't tell |
| Sheu et al | 2012 | Yes | Yes | Yes | Yes | Yes | Can't tell | Can't tell |
| Steeb et al | 2020 | Yes | Yes | Yes | Yes | Yes | Can't tell | Yes |
| Wilby et al | 2015 | Yes | Yes | Yes | Yes | Can't tell | Can't tell | Can't tell |
| Gibson and White | 2019 | Yes | Yes | Yes | Yes | Can't tell | Can't tell | Can't tell |
| Hawala-Druy & Hill | 2012 | Yes | Yes | Yes | Can't tell | Can't tell | Can't tell | Can't tell |
| Aspden et al | 2016 | Yes | Yes | Yes | Yes | Yes | Yes | Can't tell |
| Hasan et al | 2017 | Yes | Yes | Yes | Can't tell | Can't tell | Can't tell | Can't tell |

**Supplementary material 4: GREET results**

| **Studies** | **1** | **2** | **3** | **4** | **5** | **6** | **7** | **8** | **9** | **10** | **11** | **12** | **13** | **14** | **15** | **16** | **17** | **Compliant items** | **% compliance** |
| --- | --- | --- | --- | --- | --- | --- | --- | --- | --- | --- | --- | --- | --- | --- | --- | --- | --- | --- | --- |
| Strelow et al., 2021 | x | x | x |  |  | x |  |  |  | x |  |  |  |  |  |  |  | 5 | 29.4 |
| Nebergall et al., 2021 |  |  | x |  |  | x | x |  |  |  | x |  |  | x |  |  |  | 6 | 35.3 |
| Bailey et al., 2021 | x |  | x |  | x | x |  | x | x | x | x | x |  |  | x |  |  | 10 | 58.8 |
| Butler et al., 2020 | x | x |  |  |  | x |  |  |  | x | x |  |  |  |  |  |  | 5 | 29.4 |
| Dushenkov et al., 2020 | x | x | x |  |  | x |  | x |  |  | x |  |  |  |  |  |  | 6 | 35.3 |
| Min et al., 2020 | x | x | x |  |  | x |  | x | x | x | x |  |  |  | x |  |  | 9 | 52.9 |
| Boylan et al., 2020 | x | x |  |  | x | x |  |  |  | x | x |  |  |  |  |  |  | 6 | 35.3 |
| Diaz-Cruz & Hagan, 2020 | x | x | x |  |  | x |  | x | x |  | x | x |  |  |  |  |  | 8 | 47.1 |
| Chen et al., 2020 | x | x |  |  |  | x |  |  | x | x | x | x |  |  | x |  |  | 8 | 47.1 |
| Mueller, 2017 | x | x | x |  | x | x |  | x | x | x | x | x |  |  | x |  |  | 11 | 64.7 |
| Rovers et al., 2020 | x | x |  |  |  |  |  | x |  | x |  |  |  |  |  |  |  | 4 | 23.5 |
| Arif et al., 2017 | x | x | x |  |  | x |  |  | x | x | x | x |  |  | x |  |  | 9 | 52.9 |
| Hasan et al., 2017 | x | x | x |  | x | x |  | x | x | x | x | x |  |  |  |  |  | 10 | 58.8 |
| Sheu etal., 2012 | x | x |  |  |  | x |  | x | x | x | x | x |  |  | x |  |  | 9 | 52.9 |
| Durand et al., 2012 | x | x | x |  |  | x |  | x | x |  | x | x |  |  |  |  |  | 8 | 47.1 |
| Hawala-Druy & Hill, 2012 | x | x | x | x |  | x |  |  | x |  |  | x |  |  |  |  |  | 7 | 41.2 |
| Werremeyer & Skoy, 2012 | x | x | x | x |  | x |  | x | x | x | x | x |  |  | x |  |  | 11 | 64.7 |
| Arif et al., 2019 | x | x |  |  |  | x |  | x | x | x |  |  |  |  |  |  |  | 6 | 35.3 |
| Scott et al., 2019 | x |  | x |  |  | x |  | x | x | x |  |  |  |  | x |  |  | 7 | 41.2 |
| Aspaden et al., 2016 | x | x |  |  |  | x |  |  | x | x | x | x |  |  | x |  |  | 8 | 47.1 |
| Clarke et al., 2016 | x | x |  |  |  | x |  | x | x | x |  | x |  |  |  |  |  | 7 | 41.2 |
| Heffernan et al., 2013 | x | x |  |  |  | x |  | x | x | x | x |  |  |  | x |  |  | 8 | 47.1 |
| Ostroff et al., 2018 | x | x |  |  |  | x |  | x | x |  | x | x |  |  |  |  |  | 7 | 41.2 |
| Prescott & Nobel, 2019 | x | x | x | x |  | x |  | x | x | x | x | x |  |  |  |  |  | 10 | 58.8 |
| Schellhase et al., 2013 | x | x | x |  | x | x |  | x | x | x |  | x |  | x | x |  |  | 11 | 64.7 |
| Liu et al., 2015 | x | x | x |  | x | x |  |  | x |  |  |  |  |  | x |  |  | 7 | 41.2 |
| Wilby et al., 2015 | x | x | x |  |  | x |  | x | x | x | x | x |  |  | x |  |  | 10 | 58.8 |
| Cailor et al., 2015 | x | x | x |  | x | x |  |  | x | x | x | x |  |  |  |  |  | 9 | 52.9 |
| Newsome et al., 2018 | x | x | x |  | x | x |  | x | x |  | x | x |  |  | x |  |  | 10 | 58.8 |
| Parkhill et al., 2014 | x | x | x |  |  | x |  | x | x | x | x | x |  |  | x |  |  | 10 | 58.8 |
| Wietholter et al., 2014 | x |  | x |  | x | x |  | x | x | x |  |  | x | x |  |  |  | 9 | 52.9 |
| Crawford et al., 2016 | x | x |  |  |  | x |  |  | x | x | x | x |  |  | x |  |  | 8 | 47.1 |
| McKennon et al., 2018 | x | x | x |  | x | x |  | x | x | x |  |  |  |  | x |  |  | 9 | 52.9 |
| Cooper et al., 2014 | x | x |  |  |  | x |  |  | x | x | x | x |  |  | x |  |  | 8 | 47.1 |
| Echeverri & Dise, 2017 | x | x |  |  | x | x |  |  | x | x |  | x |  |  | x |  |  | 8 | 47.1 |
| Haack & Phillips, 2012 | x | x | x |  | x | x |  | x | x | x | x | x |  |  | x |  |  | 11 | 64.7 |
| Knockel et al., 2019 | x | x | x |  | x | x | x | x | x | x | x | x |  |  | x |  |  | 12 | 70.6 |
| Leach et al., 2019 | x | x | x |  | x | x |  |  | x | x | x | x |  |  | x |  |  | 10 | 58.8 |
| Thomason et al., 2013 | x |  |  |  |  | x | x | x | x |  |  |  |  |  | x |  |  | 6 | 35.3 |
| Steeb et al., 2020 | x | x |  |  |  | x |  |  | x | x | x |  |  |  | x |  |  | 7 | 41.2 |
| # of studies complying with item | 40 | 35 | 25 | 3 | 14 | 39 | 3 | 25 | 34 | 31 | 28 | 25 | 1 | 3 | 24 | 0 | 0 | - | - |
| % Compliance | 100 | 87.5 | 62.5 | 7.5 | 35 | 97.5 | 7.5 | 62.5 | 85 | 77.5 | 70 | 62.5 | 2.5 | 7.5 | 60 | 0 | 0 | - | - |
